# A New Biomedical Passage Retrieval Framework for Laboratory Medicine: Leveraging Domain-specific Ontology, Multilevel PRF, and Negation Differential Weighting

**DOI:** 10.1155/2018/3943417

**Published:** 2018-12-24

**Authors:** Keejun Han, Hyoeun Shim, Mun Y. Yi

**Affiliations:** ^1^Graduate School of Knowledge Service Engineering, KAIST, Daejeon, Republic of Korea; ^2^Department of Laboratory Medicine, National Cancer Center, Goyang, Republic of Korea; ^3^Graduate School of Knowledge Service Engineering, KAIST, Daejeon, Republic of Korea

## Abstract

Clinical decision support (CDS) search is performed to retrieve key medical literature that can assist the practice of medical experts by offering appropriate medical information relevant to the medical case in hand. In this paper, we present a novel CDS search framework designed for passage retrieval from biomedical textbooks in order to support clinical decision-making using laboratory test results. The framework utilizes two unique characteristics of the textual reports derived from the test results, which are syntax variation and negation information. The proposed framework consists of three components: domain ontology, index repository, and query processing engine. We first created a domain ontology to resolve syntax variation by applying the ontology to detect medical concepts from the test results with language translation. We then preprocessed and performed indexing of biomedical textbooks recommended by clinicians for passage retrieval. We finally built the query-processing engine tailored for CDS, including translation, concept detection, query expansion, pseudo-relevance feedback at the local and global levels, and ranking with differential weighting of negation information. To evaluate the effectiveness of the proposed framework, we followed the standard information retrieval evaluation procedure. An evaluation dataset was created, including 28,581 textual reports for 30 laboratory test results and 56,228 passages from widely used biomedical textbooks, recommended by clinicians. Overall, our proposed passage retrieval framework, GPRF-NEG, outperforms the baseline by 36.2, 100.5, and 69.7 percent for MRR, *R*-precision, and Precision at 5, respectively. Our study results indicate that the proposed CDS search framework specifically designed for passage retrieval of biomedical literature represents a practically viable choice for clinicians as it supports their decision-making processes by providing relevant passages extracted from the sources that they prefer to refer to, with improved performances.

## 1. Introduction

In patient health care, nearly 70 percent of clinical decisions are made using clinical laboratory test results [1]. Due to the large number of tests for a patient, containing up to 3,000 analytes, interpretations based on the laboratory results are commonly complex and error-prone, often causing catastrophic failure in clinical decision-making [2]. Various studies [3, 4] indicate that those errors are surprisingly frequent, and some of the errors (6.4–12 percent) potentially produce adverse impacts on patient care. These statistics highlight the urgent need for a proper CDS (clinical decision support) system, which refers to a system designed to assist clinicians by providing relevant and timely medical information [5]. In a modern context of information retrieval, one important function of a CDS system is to retrieve key medical information that can assist medical experts while examining laboratory test results, which is often referred to as CDS search [6].

In a clinical setting, clinicians have very limited time to search and absorb the information in need while performing medical practice [7, 8]. This time-pressured situation requires the relevant information in search to be retrieved and presented in a more succinct form such as in a short passage, rather than a whole page or document. In this study, we present a novel CDS search framework applicable to a common diagnostic context in which clinical laboratory test results are used to formulate medical practitioners' opinions and suggestions. Those laboratory test results commonly have two unique characteristics, not found in general queries: syntax variations [9] and negation indicators [10].

First, syntax variation mainly occurs from the usage of abbreviations, acronyms, and synonyms [11]. While performing the diagnosis based on clinical laboratory test results, the medical practitioners often use various abbreviations in an arbitrary manner [12, 13]. For instance, one of the laboratory tests, alpha-fetoprotein, is mostly abbreviated as AFP in the textual reports but also has variations such as AFP-L3 percent, Total AFP, and so on. These syntax variations present a serious challenge to detecting proper medical concepts from the query, which is known to be a crucial step for search performance [6]. Furthermore, we perform the query expansion process at a passage level and document level consecutively, in order to reduce the effect of query drift. At the passage-level expansion, words that co-occur with the initial query are detected as additional medical concepts; and at the document-level, relevance of the concepts is verified by performing PRF again on document corpus, which has more coverage over medical field than the passage corpus.

Second, experts in clinical laboratories spend a considerable amount of time writing clinical reports based on abnormal test results rather than on normal test results because false-negative errors cause doctors to miss the window of opportunity for proper medical treatment [14]. In fact, when the test results are normal, the user intent for CDS search is rather simple such as referring to the range values of the test. On the other hand, when clinicians request a CDS search task for finding supporting statements for abnormal test cases, their search intent becomes more complex as they often look for side effects and related diseases for the specific laboratory tests [15], implying that passages containing explanations of the abnormalities for the related tests should be ranked higher on the retrieved results. For instance, an abnormal query such as “Eosinophil has been increased” should put more weights on passages that contain such abnormal cases. To the best of our understanding, no studies have attempted to differentiate abnormal and normal cases, nor did they weight retrieved passages differently depending on the type of the cases.

In the proposed CDS search framework, a domain ontology for covering syntax variation and language translation as well as utilizing existing medical ontologies is included. Query expansion is performed at the passage and document levels to filter irrelevant concepts to be expanded. In addition, when the given query is derived from an abnormal case, the ranking process more heavily weights those passages that contain abnormal descriptions by exploiting the negation information of the query and passages. The proposed framework showed statistically significant improvements over the baseline and other compared approaches, including variants of some of the state-of-the-art methods [8, 16, 17].

In summary, contributions of this study are as follows: (1) we propose a new passage retrieval framework tailored to the clinical context in which laboratory test results are used as the basis to form medical practitioner's opinions and suggestions, (2) the framework addresses two issues that hold significance to the medical practice in clinical settings: syntax variations and negation indicators, and (3) the proposed framework is empirically assessed for its viability in clinical settings. The proposed CDS search framework has been applied to an organization for actual use, producing positive results.

## 2. Related Work

In recent years, a significant body of studies has been conducted on CDS search with regard to various resources on the Web since traditional information retrieval techniques cannot be directly applied to biomedical information retrieval due to the specific domain characteristics of the biomedical literature: [6] examined the possibility of using a longer length of query from a narrative structure. Furthermore, Koopman et al. [18] automatically generated a query from a verbose patient narrative using a query reduction approach. In this study, we focus on generating queries from laboratory test results, which are more specific than the narrative texts in describing patient conditions.

In biomedical literature retrieval, there are two areas that have been widely studied: concept extraction and query expansion. For concept extraction, manual efforts by doctor [19, 20] to choose medical terms with the Unified Medical Language Systems (UMLS) Metathesaurus concepts showed a promising performance on retrieval. Thanks to the National Library of Medicine (NLM), MetaMap [21] was then developed to automatically map biomedical text to the Unified Medical Language Systems (UMLS) Metathesaurus concepts. Currently, MetaMap is actively employed for concept detection in most of various biomedical tasks [6, 22–24]. One of the characteristics of MetaMap is that it generates variants of each phrase. The biomedical literature texts are mapped to clinical concepts with UMLS identifier tags, and additionally their synonyms, acronyms, abbreviations, and deviational variants are provided.

cTAKES [25] is also another widely used application for clinical concept extraction. cTAKES is designed for clinical narratives unlike MetaMap targeting biomedical literature. The results from NER annotator in cTAKES contain semantic types such as disease, symptom, and drug, and negation annotation. Wang and Fang [26] used a combination of cTAKES and MetaMap to detect noun phrases and conduct abbreviation expansion, respectively; and it revealed room for improvements in resolving ambiguity when identifying multiple terms in the same free text span.

Among techniques to enhance the performance of biomedical literature retrieval, query expansion is one of the most common techniques. The goal of query expansion is to find synonyms and other related terms to increase the recall of relevant documents [27]. The expansion techniques adopted in biomedical IR (information retrieval) so far are mainly classified into two categories: the use of external resources and pseudo-relevance feedback technique.

As external recourses, UMLS [24], Google [16], MediLexicon [26], and MeSH [20] are examples of lexical knowledge resources and are actively adopted to find relevant terms. Martinez et al. [28] used UMLS representations and constructed a graph of them. By employing random walk in the graph, they efficiently conducted the task of query expansion. Yu et al. [29] used explicit relevance feedback ticked by users, in order to run RankSVM [30] to re-rank the initial search list.

However, those ontologies only include some of representative vocabularies for a certain concept and do not cover all of the variations that occur frequently in a clinical setting [13]. Additionally, those ontologies only cover concepts in English; thus, some of the variations written in a different language cannot be covered [12]. As the medical queries have ambiguity and complexity, which makes the utilization of automatic translation difficult [31], a high-quality, domain-specific ontology that can deal with syntax variation and language translation is a necessity component for a CDS search framework in order to assist domain experts such as medical practitioners [11].

In acquiring implicit relevance feedback, pseudo-relevance feedback (PRF) is a fundamental technique of query expansion, usually done with initial expansion using external resources. It is well known to find potentially relevant terms by first querying the index and looking for new relevant terms from high-ranked documents [27]. The weakness of PRF is the possibility of adding less relevant terms and increasing noises. However, it is widely used because it is simple and straightforward, and it still is helpful in finding related terms not available in a resource. Most of current state-of-the art CDS search [6, 23, 24, 32, 33] studied medical information retrieval with pseudo-relevance feedback approach. They conducted comprehensive evaluations on different types of datasets; and the study clearly showed the effectiveness of PRF in biomedical literature retrieval.

Inevitably, CDS search based on PRF also suffers from a well-known problem of PRF called topic drifting, which degrades retrieval performance as the intention of the query topic could change in an unexpected direction due to erroneous extraction of the concepts to be expanded [34]. In order to reduce the effect of query drift, word embedding approach was utilized for term expansion [35] and search diversification [36]. Meanwhile in this paper, at the passage-level expansion, words that co-occur with the initial query are detected as additional medical concepts; and at the document-level, relevance of the concepts can be verified by performing PRF again on document corpus that has more coverage over the medical field than the passage corpus.

Moreover, no prior studies have proposed a framework dedicated to passage retrieval in a clinical setting. Most of the current CDS search studies [16, 17, 33] have only focused on improving the performance of retrieving relevant documents, which are usually academic journal articles available on the web such as PubMed (https://www.ncbi.nlm.nih.gov/pubmed/), an open-access site of biomedical article collection. Those CDS search approaches are not effective for passage retrieval, not to mention that some of the most useful biomedical resources that clinicians actively utilize are underrepresented in those open-access collection. Karimi et al. [37] offered a CDS search platform for IR researchers to use the unified datasets and CDS search algorithms. However, they were limited to the document corpus only. Instead, in this paper we focus on the passage retrieval from medical text books.

## 3. Methods

**Figure 1:**
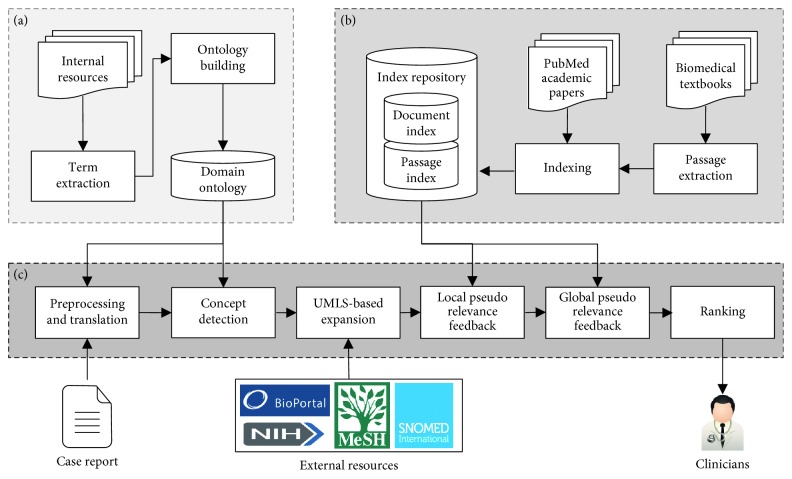
Overall architecture of the framework: (a) domain ontology creation, (b) index processing, and (c) query processing.

Figure 1 shows the overall architecture of our framework, which involves interactions among three core components: (1) domain ontology (and preceding processes needed for ontology creation), (2) index repository (and preceding processes needed for index repository), and (3) query processing engine (covering from preprocessing to ranking).

First of all, to overcome the syntax variations and language differences, the proposed framework includes a domain ontology. It is very common to see several variants of key medical concepts in practice. The variants are sometimes organizational-specific. Thus, it cannot be completely covered by public ontologies. Furthermore, as the input data used for this system are written in Korean and the passages from the books are written in English, it is necessary to build an ontology in advance to translate the initial textual report into a list of words in English. The domain ontology requires full coverage of technical terms on clinical laboratory test results and an adequate structure suited for translation and concept detection. It should also be converted to a DB schema to be stored in database for system implementation.

Second, passage-level indexes for biomedical textbooks should be made for our biomedical literature retrieval. The indexes convert the literature into a set of passages for quick access to relevant information. The reference information also should be extracted for additional data collection. In addition to indexing the biomedical textbooks, additional document collected from the web should be indexed for two main purposes: (1) the collection is used for query expansion in the proposed CDS search framework and (2) relevant articles are also returned along with the passages in our web service. Users can easily access the retrieved resources according to the types of resources (textbooks vs. articles) as those resources are provided in different tabs on the user interface.

Finally, once the domain ontology and indexes are ready, our proposed CDS search framework can be put in operation through interactions among the three components. After each sentence is translated in English, we use only medical-related terms that can be obtained via the domain ontology or external medical ontologies. We call this step concept detection (CD). Based on the concepts detected, synonyms of those concepts are added to the initial query through UMLS-based expansion (UMLSE). A well-known query expansion technique called pseudo-relevance feedback [38], which finds potentially related terms by first querying the index and then looking for other relevant terms from high ranked documents, is then performed to detect terms that are frequently used in the retrieved passages. The PRF in the proposed framework has two layers: detecting important terms at a passage level and then reweighing the terms based on their importance at document level, in order to fully utilize the advantage of having multiple corpus in the index repository. Given that the expansion is performed on the targeted passages only, we call this step local pseudo-relevance feedback (LPRF). We further extend this process to perform PRF again on a set of external medical documents collected on the web. This step is called global pseudo-relevance feedback (GPRF), as the terms are added from all medical fields. For final retrieval, a novel ranking method exploiting the importance of negation information is necessary in order to put more weight on relevant passages that contain a description of abnormal conditions for the given test results.

It should be noted that domain ontology creation and index processing are performed off-line while the other steps are performed online, in order to ensure a fast execution of query processing. In the subsequent sections, we first explain the procedure and architecture of the domain ontology creation. We then describe the index processing for targeted biomedical textbooks and external document collection for medical academic papers. Lastly, we explain the query processing component in detail.

### 3.1. Domain Ontology Creation

The quality of domain ontology, such as the coverage of domain, is a significant factor for CDS search performance [11]. More importantly, because the ontology in the present paper is also used for the translation phase, the ontology should cover all the abbreviations and words used not only in the field, but also in the specific medical organization, where this system is intended to be used in practice. In the chosen medical institution, located in South Korea, diagnosis based on clinical laboratory test results is performed under the supervision of a department of laboratory medicine that conducts liver disease-related tests, hematology-related tests, diabetes-related tests, kidney-related tests, arthritis/venereal-related tests, and cancer/thyroid-related tests. Thus, the contents within a case can be classified into diseases, tests associated with each of the diseases, and specimens required for specific tests. Those categories were defined as classes in the domain ontology. Furthermore, in order to make our framework reproducible by other researchers, existing external medical ontologies such as UMLS and KOSTOM (https://www.hins.or.kr/cmm/main/mainPage.do) were connected to our ontology by including their unique identifiers of the instances (i.e., terms), if the term co-occurs both in our ontology and in the external ontologies.

**Figure 2:**
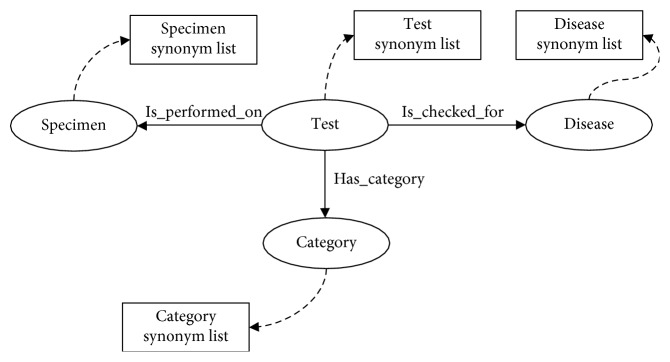
Ontology classes.

We simplified the design of the domain ontology to ensure fast access to the ontology from the system, as shown in Figure 2. The class named “Test” refers to a clinical laboratory test component for reporting a patient's health status, indicating that it connects every class within the domain ontology. For instance, an amylase test is conducted on serum to examine whether or not the patient has a fructosuria. The other classes such as “Specimen,” “Category,” and “Disease” were used to define properties of a corresponding part. “Specimen” was used to define which specimen is used to perform the given test, such as serum or urine. “Category” was used to define which category the given test belongs to. Finally, “Disease” was for disease information such as the disease name and the body part the disease affects.

We extracted all terms from the resources such as in-house test reference manuals, hardcopies of healthcare reports, and other documents available in the targeted medical organization. After extraction, mapping each term to its corresponding class type was conducted to create instances. While grouping, instances that had the same meaning were then revisited to make a lexicon. In a group, one term was selected as a representative, and the others were allocated to that instance, defined as lexicon relationship. For instance, Table 1 presents multilingual (i.e., Korean and English) synonyms of some representative instances.

The final step for the domain ontology creation was manual inspection of the ontology. In this step, field experts checked the validity of the instances and the relationships among them. In addition, terms that are not used in practice, but that can be used in queries, were added to the ontology such as the name of collaborating hospitals.

After all the steps were completed, we obtained an ontology that consisted of 4 classes, 4 properties, 668 instances, and 1,961 synonyms in the lexicons. Other than the information defined in the ontology, the instances can be further extended to external ontologies if they exist in the selected external ontologies by matching the unique identifier. For instance, “amylase,” which is an instance in the Test class can be extended to external UMLS ontology since it has its own UMLS code (C0002712) in the created ontology. It should be noted that we only used terms defined in the ontology for the present study, although the ontology contains more useful information such as relations. The ontology is publicly available to other researchers for other applications (http://kirc.kaist.ac.kr/datasets/).

### 3.2. Index Processing

We employed biomedical textbooks for passage retrieval. The length of a passage can either be a sentence or a paragraph depending on the cases. As reported in [8], medical practitioners prefer to read a paragraph where relevant information is contained in a quickly digestible form rather than the entire text page. Therefore, we focused on retrieving a set of candidate paragraphs as passages.

**Figure 3:**
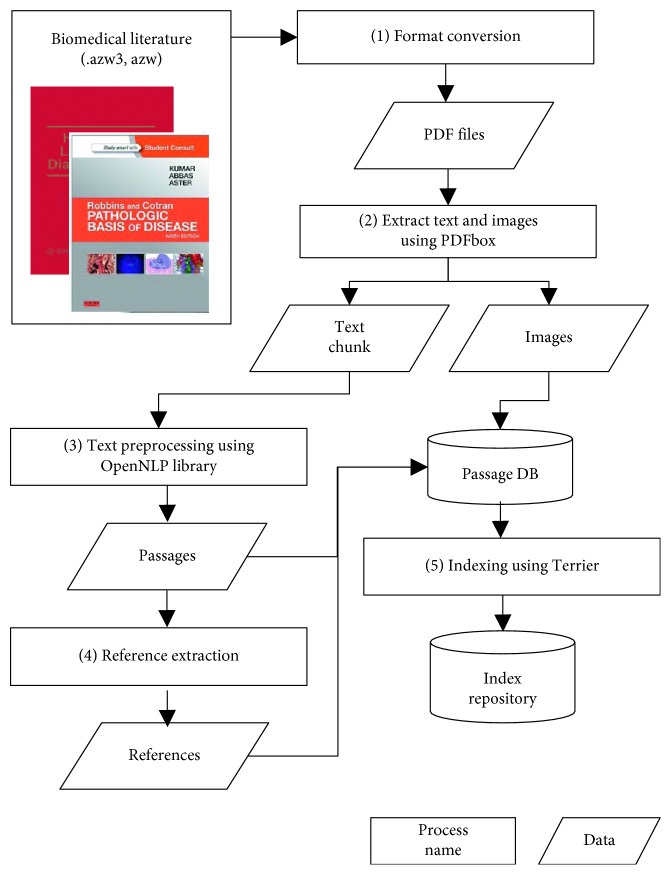
Biomedical literature indexing process.

First, we processed three eminent biomedical textbooks as they were commonly recommended by multiple clinicians who are currently making diagnostic decisions.  Figure 3 shows the overall procedure of obtaining a set of passages from the books. Before text and image extraction, transformation from the specific format of the literature to plain text is performed for further processing.

Based on the converted version of the literature, we extracted metadata including the titles, authors, chapters, references, table captions, contents, and images from the books by using PDFBox (https://pdfbox.apache.org/). The extracted images were shown to the reader when the corresponding passage was clicked. We then extracted all the paragraphs and sentences from a consecutive list of text blocks by using Stanford NLP [39]. We discarded those paragraphs with the total number of sentences less than two because they are more likely to be titles or headings. Reference entries were then extracted if they matched the format predefined to be reference section—reference header expressions such as “References,” “Bibliography,” “References and Notes,” or the end of the chapters. All of the attributes were extracted automatically, followed by manual inspection performed to verify the extraction.  Table 2 presents descriptive statistics of the medical literature we used for the passage retrieval. Recommended by the clinicians, we chose three textbooks where those titles were abbreviated as follows: HCDMLM2017 [40], PGDT2003 [41], and RCPBD2014 [42]. It is noted that there is no image for PGDT2003 [41] because it is a pocket book formatted as a consecutive list of tables without an image.

Lastly, indexing was conducted for the paragraphs as passages. Since index time, space, and storage are significant factors for the practical usage of the proposed framework, we used Terrior (http://terrier.org/) among candidate open-source search engines. It provides effective indexing creation and fast access for the passages with inbuilt query expansion techniques. If a passage contains images or tables, links to such resources were indexed along with the passages.

Other than the chosen biomedical literature, indexing the additional document collection was performed for use in the query expansion process in the proposed framework. The collection was the open-access subset of PubMed Central (PMC) (https://www.ncbi.nlm.nih.gov/pmc/). PMC is an online digital library of freely available full-text biomedical literature. The snapshot contains 733,138 articles. The full text of each article is represented as an XML file. Images and other materials were also available. Each article in the collection has a unique identifier (PMCID) within its XML file. To index the documents, we developed a simple java program to extract contents from the XML files and used Terrior to index the abstracts and contents to be used for query expansion.

#### 3.2.1. Query Processing

In this subsection, we introduce IR techniques including query expansion and ranking methods we incorporated into the proposed framework. For concept detection, we use a domain-specific tool, MetaMap [21] to detect medical-related terms. Synonyms of the terms are added in the query expansion process using our domain and external ontologies. We also introduce a refined version of pseudo-relevance feedback (PRF) on initially retrieved passages and external medical documents. It should be noted that those steps are the state-of-the-art techniques for query expansion. Finally, typology-aware ranking method is presented to fully utilize negation information from case reports based on laboratory test results.

*(1) Preprocessing and Translation*. When an initial case (in JSON—JavaScript Object Notation, a lightweight data interchange format) is inputted, the “report-text” header is identified. The texts in the header are then split into words or symbols called tokens. The texts are written in Korean; thus, morphological analysis for Korean tokens is additionally required. To do so, an open-source Korean morphological analyzer, Komoran (http://www.shineware.co.kr/products/komoran/), is used to detect only nouns from the test results. For English terms, uppercase letters are converted to lowercase letters, if they have any. Those terms are then translated into English by mapping each Korean term to a corresponding representative English term through its lexicon relationship previously defined in the ontology. If there are no corresponding English terms, those Korean terms are discarded.

*(2) Concept Detection (CD)*. This step detects a list of concepts from the terms obtained in the previous step. The domain ontology includes synonym terms for test, disease, category, and specimen that are used to detect initial concepts. Thanks to having representative terms for concepts in the ontology, all the detected concepts can be changed to its representative concepts. Then, MetaMap [21], which is widely used for concept detection in various biomedical retrieval tasks [22–24], is used to annotate each concept to its respective Unified Medical Language System (UMLS) concept. As UMLS has more than 100 semantic types, our observation indicated that mapping should be restricted to the following types: Disease or Syndrome (T047), Sign or Symptom (T184), Pathologic Function (T039), Diagnostic Procedure (T060), Anatomical Abnormality (T190), Laboratory Procedure (T059), Pharmacological Substance (T121), Neoplastic Process (T191), and Therapeutic or Preventive Procedure (T061). The initial query is then reformulated by removing all the terms that did not have any mapping to UMLS concepts.

*(3) UMLS-Based Expansion (UMLSE)*. Recent CDS search models have indicated that query expansion with Unified Medical Language System (UMLS) concepts can significantly impact the general performance of biomedical literature retrieval [28, 29, 32]. Thus, we also utilize the external medical resources in order to expand the initial list of concepts in the query. In this present study, for all the mapped UMLS concepts from the previous step, synonyms are extracted using BioPortal (https://bioportal.bioontology.org/), which consists of 586 medical ontologies including some popular ones such as MeSH and SNOMED. BioPortal offers a web API service for browsing ontologies where synonyms simply can be obtained by querying each of the mapped concepts. Note that the expanded terms are set to have lower weights, each with the weight of 0.5, than the terms in the mapped concepts.

*(4) Local Pseudo-Relevance Feedback (LPRF)*. One limitation of UMLSE is that only synonyms are added to the query but not the terms that are closely related to the original query. For instance, because “ketone” and “diabetes” are highly related in the medical field, detecting this latent information rather than only expanding the synonyms of the two terms would have a positive effect on retrieval performance. Thus, for a given query, pseudo-relevance feedback (PRF) [38], which retrieves initial articles, extracts terms from the top *N* articles, and then reapplies the expanded query for final retrieval, is performed to gather additional terms. More specifically, it obtains candidate terms that are copresented within a passage-distance level from the top *k* retrieved passages. As PRF only explored locally stored corpus and did not consider external resources, we call this step local pseudo-relevance feedback (LPRF).

We adjusted the “IDF Query Expansion” method proposed in [32] to fit our experiment by calculating a boosting coefficient for each term in the expanded query as follows: the algorithm tokenizes the top *k* passages retrieved for query *Q*. It then builds the root set *R*_*Q*_, which consists of the union of the set containing all the terms in *Q* and the set of all the terms in the retrieved passages for *Q*. For each term *t*_*j*_ ∈ *R*_*Q*_, the weight of *t*_*j*_ is obtained as follows:

(1) $$\begin{array}{c} S_{l}\left(t_{j}\right)=\log\;\left(10+S_{t}\left(t_{j}\right)\right), \end{array}$$

(2) $$\begin{array}{c} S_{t}\left(t_{j}\right)=\alpha \cdot I_{Q}\left(t_{j}\right)\cdot \frac{{\text{tf}}_{j}}{N}+\frac{\beta }{k}\overset{k}{\underset{i=1}{\sum }}I_{P_{i}}\left(t_{j}\right)\cdot {\text{ipf}}_{j}, \end{array}$$

where *t*_*j*_ is the *j*-th term in the top *k* passages, tf_*j*_ is the number of *t*_*j*_ in the root set *R*_*Q*_, *N* is the number of terms in the top *k* passages, *I*_*Q*_(*t*_*j*_) is an indicator of the presence of term *t*_*j*_ in *Q*, *I*_*P*_*i*__(*t*_*j*_) is an indicator of the presence of term *t*_*j*_ in the passage *P*_*i*_, and ipf_*j*_ is the inverse passage frequency of the *j*-th term in the top *k* passages. Finally, *α* and *β* are smoothing factors. We set the parameters to be *α* = 2, *β* = 0.75, and *k* = 5 as suggested in [32].

#### 3.2.2. Global Pseudo-Relevance Feedback (GPRF)

We further extend the framework to reflect broader clinical domain knowledge (medical terms). Global pseudo-relevance feedback (GPRF) is similar to LPRF. The only difference is that it performs on a different corpus, which is a set of PMC documents. It retrieves the top *k* documents, builds the root set *R*_*Q*_ of the query, calculates scores for each term in the root set using equation (2), and then normalizes the score using equation (1). Let us denote the score as *S*_*g*_(*t*_*j*_). By doing so, unlike UMLSE that adds synonyms only from the predefined ontologies, GPRF can discover latent terms from both document and passage corpora. Then the final term score *S*_prf_ is calculated as folows:

(3) $$\begin{array}{c} S_{\text{prf}}\left(t_{j}\right)=\lambda \cdot S_{l}\left(t_{j}\right)+\left(1-\lambda \right)\cdot S_{g}\left(t_{j}\right), \end{array}$$

where the parameter *λ* is set to 0.65, which is the best performing value observed in the experiment. Once all the weights have been determined, the terms in *R*_*Q*_ are ranked by their score, the top *m* terms not in the original query are added to *Q*, and *m* is set to be 35 for our experiment. We adapted Terrior to execute the LPRF and GPRF as it provides a customizable function of PRF. Lastly, each term in the reformulated query is then used for final passage retrieval.

#### 3.2.3. Ranking

We also employ negation information of the concepts detected in the retrieved passages. Our negation list includes expressions such as “normal,” “negative,” “no,” and “not,” which indicate the absence of the problem associated with the concept.  Table 3 shows some example sentences of normal and abnormal cases where its concepts are examined by NegEx (http://code.google.com/p/negex/) for negation status. In Table 3, it is notable that a concept is negated when a case is normal. Meanwhile, the concept is affirmed when the case is abnormal. This implies that, to focus on false-negative error prevention, the ranking function should weight more passages that contain affirmed concepts for abnormal cases.

Before differential weighting based on the polarity of query cases, we first attach “no-” prefix to the terms expanded from concepts which are negation-necessary in *Q*. For instance, in the query text “AFP is normal,” the expanded terms from the “AFP″ through UMLSE step are negated by attaching “no-” prefix. It should be noted that the original term, “AFP,” is not changed to prevent original query intent. The purpose of the prefix attachment is to more favor latent terms, which are discovered through GPRF, over unnecessary synonyms of the negated concept when the query is a normal case. Those queries are then used to retrieve initial relevant passages.

We adopted Terrior to run multiple IR models including TF-IDF, BM25, and LM (Language Model). For LM, we used Bayesian smoothing using the Dirichlet Prior with the default value of parameter (*μ* = 2500) on Terrior. Before combining, 0–1 normalization (https://en.wikipedia.org/wiki/Feature_scaling) was conducted on each score. A score of passage *P*_*i*_ for query *Q*, denoted as *S*(*P*_*i*_), is calculated by combining the scores from the IR models used as follows:

(4) $$\begin{array}{c} S\left(P_{i}\right)=\underset{N}{\sum }\frac{S\left(j,k\right)}{\sum_{M}S\left(j,k\right)}, \end{array}$$

where *S*(*j*, *k*) is the score of passage *P*_*i*_ using IR model *F*_*k*_, *N* is the total number of used IR model, and *M* is the total number of retrieved passages. Note that *N* and *M* are set as 3 and 100, respectively. Based on the negation information for original query text *Q*, the score *S*(*P*_*i*_) is finally weighted as follows:

(5) $$\begin{array}{c} \text{ABN}\left(Q\right)=\left\{\begin{array}{cc} 1, & {\text{AFFIRMED}}_{t}\geq {\text{NEGATED}}_{t},\\ 0, & {\text{AFFIRMED}}_{t}<{\text{NEGATED}}_{t}, \end{array}\right. \end{array}$$

(6) $$\begin{array}{c} S\left(P_{i}\right)=S\left(P_{i}\right)+\gamma \cdot \left\{\text{ABN}\left(Q\right)\cdot \left(1+\frac{{\text{AFFIRMED}}_{i}}{{\text{AFFIRMED}}_{i}+{\text{NEGATED}}_{i}}\right)\right\}, \end{array}$$

where NEGATED_*t*_ and AFFIRMED_*t*_ indicate the number of negated concepts and not negated concepts (i.e., affirmed) for a given original query text *Q*, respectively. Meanwhile, NEGATED_*i*_ and AFFIRMED_*i*_ indicate the number of negated concepts and affirmed concepts for a passage *P*_*i*_, respectively. *γ* is a boosting factor. This is a simplistic but effective approach to weight passages that are likely to contain descriptions about abnormal cases for clinical laboratory test results in *Q*. We then rank the passages based on their score in the descending order and return the results to the clinicians to support their decision-making.

## 4. Results

In this section, we first introduce ground-truth dataset we have created for the experiment as well as the metrics and compared methods. We then report retrieval effectiveness of the proposed framework followed by parameter sensitivity results. Lastly, we introduce a web-based service of the proposed framework installed on the medical organization to ensure the feasibility of the framework.

### 4.1. Experimental Setup

Currently, there is no appropriate benchmark dataset containing both clinical laboratory test results and biomedical textbooks for passage retrieval that could be used to evaluate the effectiveness of the proposed CDS search framework. NIST's TREC [27] provides 30 medical case reports as a query list and biomedical literature corpus since 2014. It is the most relevant dataset to the present study, but the corpus is a list of academic research papers, which is neither biomedical textbooks nor passages. Thus, in this paper, an alternative dataset was constructed for our experiment including 30 textual reports (including laboratory test results) and passages from three eminent biomedical textbooks recommended by clinicians.

Table 4 presents a full list of reports, used as queries to assess the proposed framework, containing descriptions of patients and its number of syntax variations. Those textual reports were obtained from the Department of Laboratory Medicine at Seegene Medical Foundation (SMF) (https://www.seegenemedical.com/eng/index.jsp), located in South Korea. The number of syntax variation for each textual report was obtained by examining the reference values for the corresponding tests. For instance, a textual report “AFP-L3 percent is normal” was considered as a variant of normal result on alpha-fetoprotein laboratory test, if the reference values connected to the report were within the normal range. Given a case report, our goal was to retrieve passages that can help a clinician to diagnose patients. To create a benchmark dataset for the evaluation, we have extracted 15 normal cases and 15 abnormal cases in the descending order of frequency, respectively, with a total of 30 cases. The reason of categorizing the cases is to see if there is a performance difference between normal and abnormal cases. In practice, abnormal cases should not be overlooked when making a medical decision, as it may lead to critical misdiagnosis. Therefore, a robust CDS search framework should not have significant performance difference between normal and abnormal cases. In total, 28,581 variants of textual reports from 30 cases were used for the experiment.

To compare performances, five retrieval models (UMLSE without domain ontology as baseline, UMLSE, LPRF, GPRF, and GPRF-NEG) were built for our experiment as shown in Table 5. Based on the fundamental NLP techniques to capture nouns from the reports, the baseline model and UMLSE were designed to observe the effect of domain ontology for concept detection. After synonym expansion by utilizing external resources on the web, LPRF and GPRF were differentiated in query expansion to measure the impact of performing PRF at the local and global level corpus. Lastly, GPRF-NEG includes the domain ontology, multilevel PRF, and negation differential weighting to assess the overall performance of the proposed framework in its entirety.

Our evaluation framework emulated standard TREC evaluation procedures for ad hoc retrieval tasks [27]. Those models were submitted individual runs per topic, each run consisting of a sorted list of up to 500 passages per topic. Each topic was judged by at least two assessors to ensure the reliability of the assessment. For the baseline, we detected nouns only as concepts by applying fundamental NLP techniques and conducted expansion based on the nouns. The assessment was performed by 20 assessors, most of whom were medical professionals from various institutions in South Korea. The medical professionals consisted of clinical pathologists and nurses. For their convenience, we developed a web service of assessing passages, which also can be accessed via mobile devices. Each assessor completed three queries, and every query was performed by at least two assessors, so as to measure interrater agreement. To avoid potential biases from the assessors, the passages were sorted randomly before they were presented to the assessors.

Given a query, assessors judged passages as either “Definitely Relevant,” “Possibly Relevant,” or “Not Relevant.” To be “Definitely Relevant,” a passage should provide information relevant to the particular patient described in the topic (i.e., case report). The information would provide diagnosis, test, and treatment of the patient described in the topic. On the other hand, a passage is judged “Possibly Relevant,” if an assessor believed it was not immediately informative on its own, but that it may be relevant in the context of a broader literature review. Finally, a passage is judged “Not Relevant” if they did not provide any information relevant to the particular aspect of the patient described in the topic. For the dataset, the interannotator agreement was 0.63, which indicates substantial agreement [43]. Finally, for each query, we obtained on average of 236.8, 62.38, and 78.38 number of passages for “Not Relevant,” “Possibly Relevant,” and “Definitely Relevant,” respectively. For the CDS search evaluation, the possibly relevant and definitely relevant sets were conflated into a single set (“Relevant”).

We used precision at *N*, Normalized Discounted Cumulative Gain (nDCG), *R*-precision, and Mean Reciprocal Rank (MRR) to evaluate the effectiveness of CDS search. First, precision at *N* passages (P@N) measures how many relevant passages are retrieved in the *N* passages. Similarly, *R*-precision is defined as follows:

(7) $$\begin{array}{c} R‐\text{precision}=\frac{r}{R}, \end{array}$$

where *r* is the number of relevant passages at *R* and *R* is the total number of relevant passages for a given query.

Second, nDCG measures the average performance of a CDS search's ranking scheme. It assigns more weights on highly ranked passages as follows:

(8) $$\begin{array}{c} \text{DCG}\left(p\right)=\left\{\begin{array}{cc} G\left(1\right), & \text{if}\;\;p=1,\\ \text{DCG}\left(p-1\right)+\frac{G\left(p\right)}{\log\left(p\right)}, & \text{otherwise,} \end{array}\right. \end{array}$$

where *p* indicates the rank position, DCG(*p*) denotes the DCG value accumulated at the rank position *p*, and *G*(*p*) denotes the gain value and its value is fixed at 1 if the passage is relevant at *p*. Then, the final DCG score is normalized from 0 to 1 by IDCG, which is the best possible DCG value as follows:

(9) $$\begin{array}{c} \text{NDCG}\left(p\right)=\frac{\text{DCG}\left(p\right)}{\text{IDCG}\left(p\right)}. \end{array}$$

Lastly, MRR measures how well a proposed approach rank resources. It is defined as follows:

(10) $$\begin{array}{c} \text{MRR}=\frac{1}{n}\times \overset{n}{\underset{i=1}{\sum }}\frac{1}{{\text{POS}}_{i}}, \end{array}$$

where *n* is the number of queries and POS_*i*_ is the position of the target resource in the result list. The larger MRR is, the faster for the user to access the resources in the list.

Compared methods were baseline and three of the state-of-the art models [8, 16, 17], which are similar to UMLSE, LPRF, and GPRF. It should be noted that those three state-of-the-art models were proven to be one of the best performing models for CDS search [27].

### 4.2. Effectiveness Evaluation

**Figure 4:**
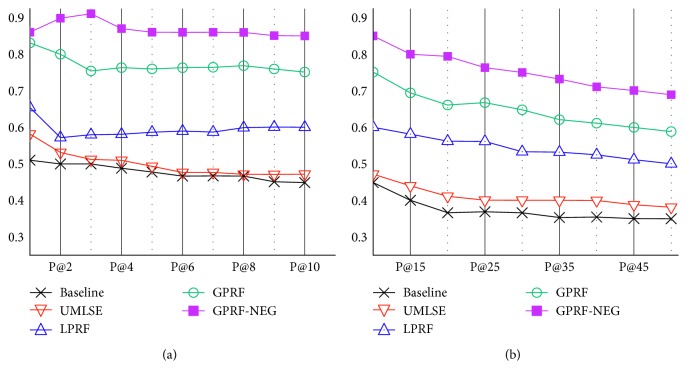
Points of precision for each method. (a) Precision from 1 to 10; (b) Precision from 10 to 50.

CDS search focuses on precision rather than recall as the framework aims to help clinicians determine the next action in care of a patient.  Figure 4 presents the precision performance at the first ten points, and then up to fifty points, in order to observe the overall precision performance tendency. We focused on precision at five passages retrieved (P@5) for the main metric, as the performance of each method was consistent up to fifty points and, more importantly, clinicians were likely to read the top retrieved passages in practice. For this reason, Table 6 presents the performance results based on MRR, *R*-precision, and P@5 to assess the quality of the proposed framework. We further analyzed the tendency of precision as the precision goes from one to fifty. It is worth mentioning that we used a paired *t*-test to measure whether the difference between any two methods was statically significant (*p* < 0.01).

In general, the baseline performed very poorly, implying that the general approach of exploiting nouns as concepts themselves holds a clear limitation to be used solely in CDS search. More importantly, the baseline fails to detect local language as concepts—another explanation of performance degradation occurring in the absence of a domain ontology. As a proof, UMLSE, which includes concept detection (CD) with synonym-oriented expansion based on those concepts, showed significant improved performance over the baseline by 6.9, 7.1, and 3.2 percent in MRR, *R*-precision, and P@5, respectively.

LPRF outperformed the baseline by 13.0, 43.2, and 22.8 percent in MRR, *R*-precision, and P@5, respectively. It also showed a statistically significant improvement over UMLSE by 6.9, 33.6, and 18.9 percent for each metric, showing that the inclusion of latent information contributed to more effective CDS search. For example, for the query “the uric acid concentration has increased,” LPRF discovered “gout” as a relevant term from the initially retrieved passages, which is, in fact, the disease diagnosed by clinicians based on the given query.

GPRF further achieved a substantially better performance over the baseline. It outperformed the baseline by 30.5, 45.4, and 59.1 percent in MRR, *R*-precision, and P@5, respectively. Furthermore, it achieved a statistically significant improvement over LPRF by 15.5 and 29.5 percent for MRR and P@5, respectively, indicating that utilizing latent information based on the external document corpus positively contributes to search performance. The results also highlight the limited coverage of the portion of the latent information existing in the passage corpus compared to the external document corpus.

We achieved the most noteworthy performance by using the GPRF-NEG. It outperformed the baseline by 36.2, 100.5, and 69.7 percent in MRR, *R*-precision, and P@5, respectively. Furthermore, it outperformed GPRF by 4.3, 37.9, and 14.0 percent in MRR, *R*-precision, and P@5, respectively. The results indicate that the inclusion of negation in the ranking function led to a more effective CDS search. For instance, for the query “Eosinophil has been increased,” GPRF failed to detect “increased” as a potentially relevant term for retrieval, which was the key identifier in diagnosing the “parasite infections” or “allergic disease.”

**Figure 5:**
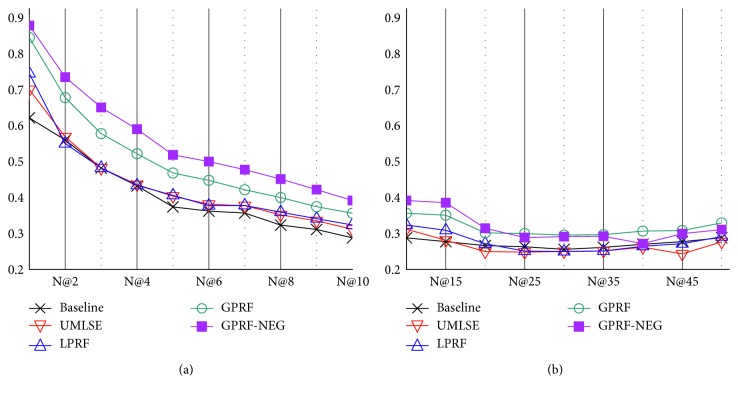
nDCG results from 1 to 50. (a) nDCG from 1 to 10; (b) nDCG from 10 to 50.

The analysis based on the nDCG throughout the first ten points (Figure 5(a)) showed that the proposed method, GPRF-NEG, consistently outperformed the baseline in nDCG@1, nDCG@5, and nDCG@10 by 41.3, 38.7, and 36.1 percent, respectively. Because of the limited size of the relevance judgement results, it showed only marginal difference between N@10 and N@20 and no significant difference from N@20 (Figure 5(b)). The rest of the improvement rates are presented in Table 7.

On the other hand, the ranking scheme is also another significant factor that influences search effectiveness. To ensure the quality of the proposed ranking scheme that combines the three aforementioned ranking schemes in conjunction with negation differential weighting, we compared the nDCG performance of each scheme on abnormal cases.  Table 8 shows that the combination of the different ranking schemes achieved a significant improvement over each of the scheme. It also achieved the best performance when the negation information was in use.

We further investigated the performance difference based on precision and nDCG results between GPRF and GPRF-NEG by dividing the cases into normal and abnormal, in order to verify the impact of utilizing negation information in the query and passages. We specifically focused on the performance gap between abnormal and normal cases to present how the gap made by different case type is alleviated by exploiting the negation information from the case report and retrieved passages.

**Figure 6:**
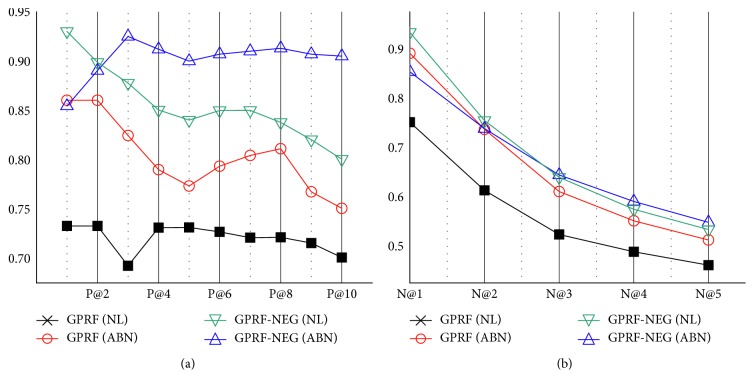
10 points of precision (a) and five points of nDCG (b) for two best performing methods (GPRF and GPRF-NEG) on normal case (denoted as NL) and abnormal case (denoted as ABN).

In terms of precision (Figure 6(a)), in the case of GPRF, the average of performance difference on the first five points of precision between normal and abnormal cases was approximately 0.104, indicating a substantially large performance gap. On the other hand, it was 0.048 on the GPRF-NEG, which was decreased by 53.07 percent, indicating that the proposed method performed better regardless of the types of case reports. It is worth noting that the performance for the normal results was also increased by utilizing the negation information although Equation (6) tends not to affect the ranking result when the case is normal; we attribute this improvement to the effect of including “no-” prefix to the negated concepts. By doing so, query filtering was processed by excluding inappropriate concepts, which were negated, from the query list. The gap was marginal in terms of nDCG (Figure 6(b)). However, the tendency was consistent that GPRF-NEG showed more condensed and higher performance on the overall nDCG points. Therefore, the substantial alleviation of the performance gap was resulted from combining two major features of case reports based on clinical laboratory test results: first, by expanding the terms that were considered important in multiple domain-specific corpus; second, by weighting the passages depending on the typology of queries.

**Figure 7:**
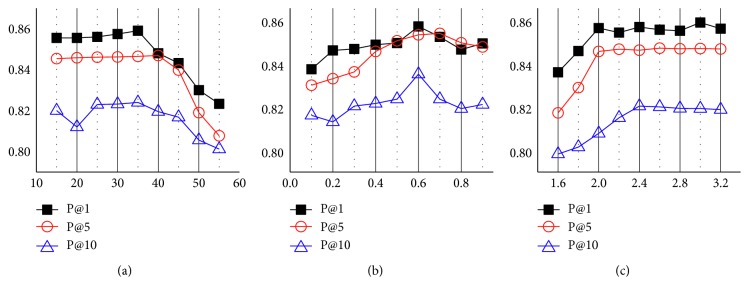
Effect of parameter values for GPRF-NEG in terms of P@1, P@3, and P@5. The best performances are achieved when (a) *m* = 35, (b) *λ* = 0.65, and (c) *γ* = 2.0.

Lastly, we observed the outcome of varying the parameters that most impacted the effectiveness of the proposed framework in terms of precision at one, five, and ten. First, Figure 7(a) presents the parameter *m*, which is the number of terms to be added for query expansion. Second, Figure 7(b) presents the parameter *λ*, which balances the PRF effect on different corpus, and Figure 7(c) presents the effect of boosting parameter *γ* to control the impact of exploiting negation information. Based on the results, the best precision performances were achieved when *m* =35, *λ* = 0.65, and *γ* = 2.0.

### 4.3. Service Implementation

**Figure 8:**
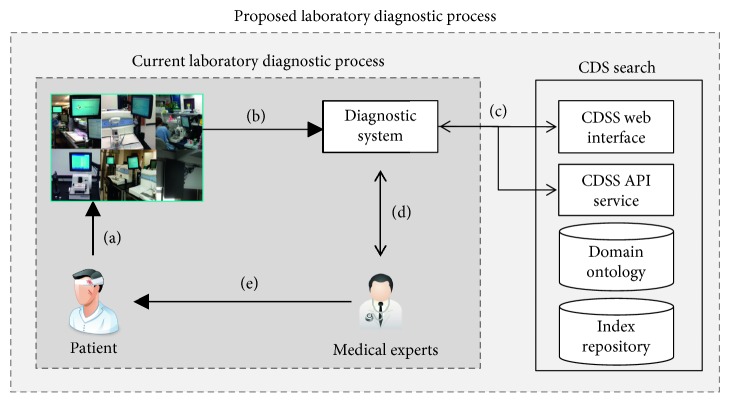
Expanded laboratory diagnostic process including the proposed CDS search framework. (a) Clinical specimens are delivered to a clinical laboratory. (b) Clinical laboratory test results are sent. (c) Interactions between the diagnostic system and CDS search framework are occurred. (d) Final decision is made based on the retrieved passages. (e) Patient report is made based on the clinical decision for patient care.

We implemented a web-based CDS search service based on the proposed framework for clinicians at Seegene Medical Foundation.  Figure 8 illustrates how the web service was added to the current laboratory diagnostic process. When the specimens of patients are delivered to a clinical laboratory (Figure 8(a)), machines in the laboratory produce test results and return them to the diagnostic system connected to the desktop computers used by clinicians (Figure 8(b)). Unlike the current laboratory diagnostic process shown in Figure 8, the proposed framework is utilized after the clinician inputs an initial textual report based on the results. In this expanded process, the report is promptly sent to the proposed CDS search framework to retrieve relevant passages via a CDS search web interface and API (Application Programming Interface), providing the search results in the JSON format (Figure 8(c)). Clinicians can confirm their initial report or look for additional relevant information by reading the retrieved passages (Figure 8(d)). Lastly, a patient report written by the medical experts is used during the consultation time for patient care (Figure 8(e)). The proposed system was installed in conjunction with the existing diagnostic system in the Department of Laboratory Medicine at Seegene Medical Foundation (SMF) (https://www.seegenemedical.com/eng/index.jsp), which runs clinical laboratory centers at multiple locations across South Korea. The service also includes APIs to return a list of relevant passages and PMC articles in JSON by inputting a textual query. Thanks to this restful coupling, database and web server for the service can be managed in different physical locations. The system was implemented based on JSP/Tomcat 8.0.

**Figure 9:**
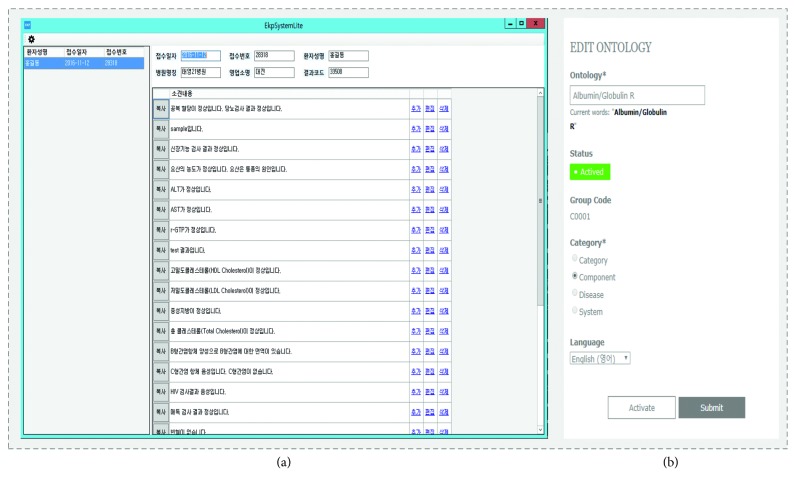
Interface snapshot for (a) new version of the diagnostic system, and (b) ontology management.

Figure 9 presents snapshots of the interface for the diagnostic system and ontology management. The proposed system was designed to work in conjunction with the existing diagnostic system rather than work as a stand-alone system, in order to minimize the learning curve associated with the new system. In Figure 9(a), on the diagnostic system, the user can activate the CDS search function in a new browsing tab by clicking the corresponding initial report. By clicking the “setup” icon on the left-top corner, the list of terms in the domain ontology can be edited by searching and browsing terms, as shown in Figure 9(b).

**Figure 10:**
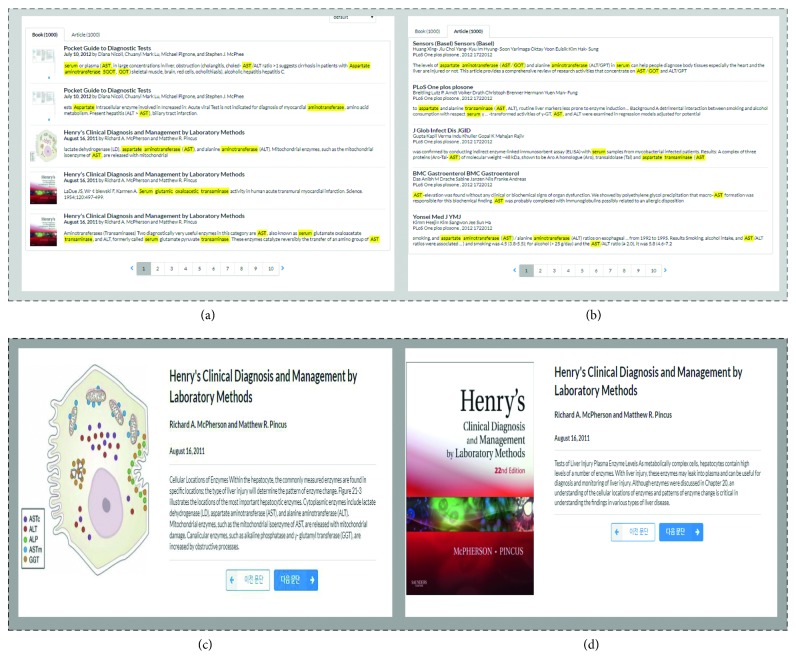
Interface snapshot for (a) a list of snippet capturing highlighted sentences for relevant passages with thumbnails, (b) a list of snippet capturing highlighted sentences from abstracts in PMC articles, (c) detailed page view, and (d) previous/next page view.

Figure 10 presents snapshots of the interface for the search results and detailed view. Figure 10(a) shows that a list of snippet for relevant passages is returned along with corresponding thumbnail images and tables. The concepts detected from the proposed framework are highlighted. If the retrieved passages contain some images or tables, its thumbnails are also given. On the other hand, Figure 10(b) shows that the “article” tab provides a list of relevant PMC articles. Each article is linked to its PMC location for browsing. Once a passage is clicked from the list, metadata such as title, author, and published date are displayed along with their detailed contents as shown in Figure 10(c). To read the previous and next passages, a clinician can click the appropriate buttons. Figure 10(d) shows the next page of the passage presented in Figure 10(c).

## 5. Discussion

We have introduced a CDS search framework, which effectively matches key terms identified from clinical laboratory test results to relevant biomedical passages. The proposed framework is distinguished from prior retrieval models commonly designed to retrieve web documents or academic journals in that the proposed framework focuses on retrieving textual contents that are in a quickly digestible form (i.e., short passages) for practical usage in a clinical setting. To effectively retrieve passages relevant to the diagnostic case in question, the framework highlights the significant roles performed by concept detection, expansion via ontologies, pseudo-relevance feedback operating at the local and global levels, and differential weighting of negation information. To empirically assess the incremental effects of those components in search performance, we have examined the five retrieval models: baseline, UMLSE, LPRF, GPRF, and GPRF-NEG. The experimental results show that each added component positively influences search performance and that the best performance is achieved by having all of these components in place. In fact, the mainstream of current biomedical literature retrieval has mostly covered the three components: concept detection, query expansion, and ranking [44]. Our findings indicate that concept detection and query expansion both get enhanced with ontologies. Also, local and global pseudo-relevance feedback and negation differential weighting are all additionally important measures that can further improve the retrieval performances of biomedical literature.

More specifically, UMLSE, which utilizes a domain ontology during concept detection process, outperformed the baseline. This finding highlights the significance of building domain-specific ontology in detecting medical concepts from the initial query. Another way of dealing with syntax variation to minimize negative effect of query drifting is to perform PRF on different corpus to verify the relevance of terms to be expanded. In the experiment, GPRF that performs PRF at different corpus, outperformed LPRF in all metrics, indicating that filtering noisy terms by applying multilevel PRF techniques positively influences retrieval performance. Lastly, all of the features including negation differential weighting contained in the proposed framework should be put together to achieve the best performance as GPRF-NEG outperformed all of the competing models in this experiment.

The proposed framework has been implemented as a web-based CDS search service in conjunction with an existing diagnostic system for actual usage in a large-size medical institution. To the best of our knowledge, this is the first study to bring CDS search into diagnostic decision-making for practical usage. In order to make the proposed framework fully tailored to the targeted specific clinical domain, we took advantages of two unique characteristics on the textual reports derived from the clinical laboratory test results.

First, we noticed that the frequent usage of abbreviation and syntax variation might lead to potential degradation of the concept detection process. Despite the studies [6, 22, 23, 26] showed some improvement for concept detection, those approaches were still inadequate to cover all of the syntax variations as they contained only a limited set of representative terms and some of their synonyms. Thus, we created a customized ontology specialized in the domain of clinical laboratory tests. The experiment results indicate that the domain ontology built by investigating domain documents and resources in cooperation with laboratory practitioners was able to effectively cover variations and abbreviations used in this clinical setting.

In dealing with syntax variation through query expansion, we also highlighted the advantage of performing a mixed use of passage-level and document-level query expansion on different corpus. Query expansion was dealt with in most of the CDS-related studies [6, 23, 24, 28, 29, 32, 33]. However, query expansion was only performed on document-level corpus as most of those studies primarily focused on document retrieval, not passage retrieval.

In the present study, because the weakness of PRF was the possibility of adding less relevant terms and increasing noise, we weighted more heavily if the terms were considered important in the corpus. For the passage-level expansion (local PRF), terms that have higher proximity to queries were expanded. Furthermore, related terms not occurred in the passages were captured through the document-level expansion (global PRF). The experiment results show that this combination of two expansion methods is highly effective for passage retrieval in filtering irrelevant terms from a list of candidate terms.

Second, we found that negation information in the query and passages should be treated with care to obtain further improvements on the retrieval as the information is a significant factor. Handling negations in CDS search has been dealt with in some studies [16, 45–47]. However, it was not directly used in the ranking function. Li et al. [16] simply pruned negated concepts during the concept detection process, and Oh and Jung [45] and Wing and Yang [47] weighted documents differently if there were polarity-matched concepts in the query and retrieved documents; however they did not treat queries differently based on its type. Wei et al. [46]'s study may be the closest to our work as it also added prefix to negated concepts so that only polarity-matched concepts would be found. However, unlike the previous studies [16, 45–47], we fully utilized negation information into our ranking function as well as the concept detection process, when a given query was supposed to be abnormal. As shown in the experiment, this approach was found to increase the number of relevant passages in the retrieved list as well as to influence the ranked list itself.

To sum up, the importance of the experiment results are threefolds. First, the study results confirm the importance of domain-specific ontology for CDS search although there already exist several medical ontologies. The results indicate that domain-specific ontology can bridge the gap between the external ontologies and the application to resolve the limitation of syntax variation and consequently to improve concept detection. Second, the study results highlight the significance of PRF performed both at the local and global levels. Through the local-level query expansion, terms that occur near the query can be captured to be expanded further. Those terms are then reweighted by its extent of medical context measured through global-level query expansion. This combinatory approach relieves the effect of query drifting by lessening the possibility of inserting noisy expanded terms into the query. Finally, although it is a simplistic approach, taking an advantage of negation information in the framework is a significant factor to achieve further improvements on passage retrieval. Thanks to its simplistic approach, the proposed re-ranking can be performed on-the-fly, implying that the actual CDS search application based on the framework still promises fast execution and no delay with the improved retrieval effectiveness.

There is room for further improvement. First, in this study, we only dealt with case reports of clinical laboratory test results. However, there are more medical tasks that the proposed framework can be applied to. Our future work aims to expand the dataset to employ as many medical fields as possible. For conducting future work, additional query sets from histopathology and passages from other medical literature are in the process of collection. Second, passage retrieval can be further improved by incorporating proximity information as it imposes a proximity constraint on the matched query terms within a passage level. One of the possible extensions for exploiting proximity is to use knowledge structure [48], which graphically visualizes key concepts and their relationships in a specific domain where nodes are concepts with their associative relationships. In [49], knowledge structure was shown to be useful for proximity-aware information retrieval by exploring abundant relationships between the concepts in a specific domain. Future research may exploit it to further improve the efficiency of the proposed framework. In [50], a bipartite graph is generated between top retrieved documents and entities extracted from the documents to measure entity importance of the candidate entities, and those importance scores were used to re-rank the initial search results. Although we focus more on query extension rather than on re-ranking, this graph-based approach can still be adapted to extract candidate terms from the knowledge structure additionally by measuring the term importance as well as co-occurrences of the terms. Finally, as our primary goal was to propose a novel framework to improve passage retrieval for CDS search, the usability of the proposed CDS service installed at the institution was not surveyed. Those formal usability tests will be performed in our future work to observe how helpful the proposed framework is for field experts through user survey and log data analysis. Notwithstanding these limitations, our study still provides important insights on designing domain-specific CDS search system that utilizes unique characteristics of the clinical settings to ensure reliable performance of the CDS search service.

## 6. Conclusion

This study shows that the proposed CDS search framework specifically designed for passage retrieval of biomedical literature enhances the passage retrieval performances, relative to extant retrieval approaches. The experimental results empirically show that the framework components of concept detection coupled with domain ontology, UMLS-based expansion, local pseudo-relevance feedback, global pseudo-relevance feedback, and ranking with negation differential weighting all have merits and add uniquely to overall performance of passage retrieval in clinical settings. In particular, our findings highlight the importance of considering unique characteristics (i.e. syntax variation and negation information) of the clinical domain in furthering CDS search performances. Additionally, our findings indicate that the pseudo-relevance feedback performed at the local and global levels both have significant effects on search. We intend to publish all the dataset that we have created including the domain ontology and ground-truth to the public so that those dataset can be used by other researchers in furthering the support of clinical decision-making.

## Figures and Tables

**Table 1:** An example of the domain ontology.

Representative term	Class name	Korean synonyms		English synonyms
Platelet	Test	혈소판		PFA, blood disk, PFT, platelet aggregation, platelet function, thrombocyte
Acute leukaemia	Disease	백혈병, 급성 백혈병		Leukaemia acute, leukaemia, acute leukaemia
Inflammation	Category	염증, 염증 관련		Inflammatory, phlogistic
Serum	Specimen	세럼, 혈청, 면역 혈청		Blood serum, sera, serum (blood), serum sickness-like reaction

**Table 2:** Descriptive statistics of biomedical literatures used for passage retrieval.

	HCDMLM2017	PGDT2003	RCPBD2014
Number of pages	5,070	652	3,269
Number of chapters	77	9	29
Number of images	1,362	—	1,445
Number of sentences	109,381	10,231	55,369
Number of paragraphs	31,456	5,051	19,721

**Table 3:** Opposite relation between negation status and case status of example sentences reported in the laboratory test results (the bold words in the sentence denotes concepts that were examined for negation status).

Sentences	Case status	Negation status
**AFP** is normal	Normal	Negated
No **anemia**	Normal	Negated
**HIV test** is negative	Normal	Negated
**Eosinophil** has been increased	Abnormal	Affirmed
**The uric acid concentration** has increased	Abnormal	Affirmed
**Bilirubin** is high	Abnormal	Affirmed

**Table 4:** A list of queries.

Case text (translated)	Type	The number of unique variants
Blood test result is normal.	Normal	3,741
No anemia.	Normal	3,150
Kidney function test result is normal	Normal	2,623
AST is normal.	Normal	2,123
Total cholesterol is normal.	Normal	2,007
Triglyceride is normal.	Normal	1,982
Syphilis test result is normal.	Normal	1,856
AFP (alpha-fetoprotein) is normal.	Normal	1,009
The concentration of uric acid is normal.	Normal	951
R-GTP is normal.	Normal	932
Fasting blood sugar level is normal.	Normal	922
HIV test result is negative.	Normal	719
Thyroid function test is normal.	Normal	321
CEA (cancer antigen) is normal	Normal	320
The rheumatoid factor (RF) is normal	Normal	288
Vitamin D (25OH-vitamin D) is deficit.	Abnormal	826
White blood cell in urine is positive.	Abnormal	598
Hemoglobin and red blood cells were detected in urine.	Abnormal	467
It corresponds to fasting blood sugar disorder. The Korean Diabetes Association has designated fasting blood sugar level 100–125 mg/dL as a fasting blood sugar disorder.	Abnormal	438
A ketone was detected in the urine.	Abnormal	434
Protein in urine is detected.	Abnormal	430
White blood cell is detected in urine.	Abnormal	410
Eosinophil has been increased.	Abnormal	395
High triglyceride.	Abnormal	325
High-density cholesterol (HDL cholesterol) has been reduced.	Abnormal	293
The uric acid concentration has increased.	Abnormal	244
Rheumatoid factor (RF) is positive.	Abnormal	207
Crystals have been found in the urine.	Abnormal	203
The high hemoglobin (HbA1c) suggests that blood glucose levels remained high for the past three to four months.	Abnormal	191
Bilirubin is high.	Abnormal	176

**Table 5:** Retrieval features used by compared retrieval models.

	Concept detection		Query expansion			Ranking function			
	NLP	Domain ontology	Synonym	Local	Global	TF-IDF	BM25	LM	Neg
Baseline	x	—	x	—	—	x	x	x	—
UMLSE	x	x	x	—	—	x	x	x	—
LPRF	x	x	x	x	—	x	x	x	—
GPRF	x	x	x	x	x	x	x	x	—
GPRF-NEG	x	x	x	x	x	x	x	x	x

**Table 6:** Evaluation results of the methods.

Method	MRR		*R*-precision		P@5	
Baseline	0.6811	—	0.3128	—	0.4777	—
UMLSE	0.7286^‡^	+6.9%	0.3351^‡^	+7.13%	0.4933^‡^	+3.2%
LPRF	0.7694^‡^	+5.6%	0.4479^‡^	+33.6%	0.5867^‡^	+18.9%
GPRF	0.8889^‡^	+15.5%	0.4549^†^	+1.5%	0.7600^‡^	+29.5%
GPRF-NEG	0.9278^‡^	+4.3%	0.6273^‡^	+37.9%	0.8667^‡^	+14.0%

^†^A significant improvement (*p* < 0.01) over the baseline. ^‡^A significant improvement over baseline and methods marked with †.

**Table 7:** nDCG results of the methods.

Method	nDCG@1		nDCG@5		nDCG@10	
Baseline	0.6211	—	0.3738	—	0.2878	—
UMLSE	0.7001^‡^	+12.72%	0.4038^‡^	+8.0%	0.3118^‡^	+8.3%
LPRF	0.7444^‡^	+6.3%	0.4059^†^	+0.5%	0.3235^‡^	+3.7%
GPRF	0.8444^‡^	+13.4%	0.4681^‡^	+15.3%	0.3563^‡^	+10.14%
GPRF-NEG	0.8778^‡^	+3.9%	0.5184^‡^	+10.7%	0.3916^‡^	+9.9%

^†^A significant improvement (*p* < 0.01) over the baseline. ^‡^A significant improvement over baseline and methods marked with †.

**Table 8:** Evaluation results by different ranking schemes on abnormal cases.

Ranking scheme	nDCG@1		nDCG@5		nDCG@10	
TF-IDF	0.8108	—	0.4418	—	0.3384	—
BM25	0.8156	+0.59%	0.4410	−0.18%	0.3476^†^	+2.72%
LM	0.8312^†^	+1.91%	0.4569^†^	+3.61%	0.3399	−2.22%
Proposed without NEG	0.8378^†^	+0.79%	0.4583^†^	+0.31%	0.3417^†^	+0.53%
Proposed with NEG	0.8483^‡^	+1.25%	0.4691^‡^	+2.36%	0.3533^†^	+3.39%

^†^A significant improvement (*p* < 0.0.1) over the baseline. ^‡^A significant improvement over baseline and methods marked with †.

## Data Availability

The datasets used to support the findings of this study are available at http://kirc.kaist.ac.kr/datasets/.
